# Aetiology, Clinical Presentation, and Outcome of Meningitis in Patients Coinfected with Human Immunodeficiency Virus and Tuberculosis

**DOI:** 10.1155/2011/180352

**Published:** 2011-12-15

**Authors:** Smita Bhagwan, Kogieleum Naidoo

**Affiliations:** Centre for the AIDS Programme of Research in South Africa (CAPRISA), Doris Duke Medical Research Institute (2nd Floor), Nelson R. Mandela School of Medicine, University of KwaZulu-Natal, Private Bag X7, Congella, Durban 4013, South Africa

## Abstract

We conducted a retrospective review of confirmed HIV-TB coinfected patients previously enrolled as part of the SAPiT study in Durban, South Africa. Patients with suspected meningitis were included in this case series. From 642 individuals, 14 episodes of meningitis in 10 patients were identified. For 8 patients, this episode of meningitis was the AIDS defining illness, with cryptococcus (9/14 episodes) and tuberculosis (3/14 episodes) as the commonest aetiological agents. The combination of headache and neck stiffness (78.6%) was the most frequent clinical presentation. Relapsing cryptococcal meningitis occurred in 3/7 patients. Mortality was 70% (7/10), with 4 deaths directly due to meningitis. In an HIV TB endemic region we identified cryptococcus followed by tuberculosis as the leading causes of meningitis. We highlight the occurrence of tuberculous meningitis in patients already receiving antituberculous therapy. The development of meningitis heralded poor outcomes, high mortality, and relapsing meningitis despite ART.

## 1. Introduction

Tuberculosis (TB) is the most common opportunistic infection in patients with Human Immunodeficiency Virus (HIV). The estimated relative risk of HIV-infected individuals developing TB is 20.6 compared to HIV uninfected, in populations with a generalized HIV epidemic [1]. 

HIV contributes significantly to the overall incidence, prevalence and poorer outcomes of meningitis. There is a predominance of chronic opportunistic meningitides in HIV-infected individuals with a higher risk of mortality and impaired cognition [2–6]. It is assumed that positive TB status would account for a greater proportion of tuberculous meningitis among HIV-TB coinfected patients as opposed to cryptococcal meningitis. However, in patients with advanced HIV infection, cryptococcus is the most common aetiology [2]. 

With increasing numbers of HIV-TB coinfected patients presenting to health facilities and high mortality related to meningitis, we aim to describe the aetiology, clinical presentation, and outcomes of meningitis in HIV-TB coinfected individuals.

## 2. Methods

We retrospectively reviewed HIV-TB coinfected patients with suspected meningitis. Patients 18 years and older, with confirmed pulmonary TB and HIV, enrolled into the SAPiT study, presenting with suspected meningitis were included in this study. The SAPiT study was a prospective randomized control trial conducted in Durban, South Africa (June 2005–July 2008), investigating the optimal timing of antiretroviral therapy (ART) initiation in patients on antituberculous therapy. All patients in the SAPiT study were sputum smear positive for mycobacteria on enrollment. They were randomly assigned to three groups, each group initiating ART at a different stage in tuberculosis therapy: within 4 weeks after the start of tuberculosis therapy (early arm), within 4 weeks after the completion of the intensive phase of tuberculosis therapy (post intensive arm), or within 4 weeks of completion of tuberculosis therapy (postcontinuation arm) [7].

Meningitis was suspected in patients who had meningeal symptoms (headache, neck stiffness, photophobia, vomiting) alone or occurring with fever, altered level of consciousness, or focal neurological signs. Data on clinical presentation, investigations, and outcomes were extracted from records at the clinical trial site and hospitals to which patients were admitted. The diagnoses of meningitis were made using a combination of recorded clinical and cerebrospinal fluid (CSF) criteria.

- Cryptococcal meningitis: diagnosed based on a positive CSF India ink stain, CSF cryptococcal antigen test or patients initiated on systemic antifungal agents with positive response.
- Tuberculous meningitis: diagnosed if *Mycobacterium tuberculosis* was isolated in the CSF. In the absence of definitive microbiologic evidence, tuberculous meningitis was diagnosed based on neurological findings in combination with typical CSF alterations (lymphocytic predominance, raised protein, reduced glucose), with exclusion of other aetiologies.
- Acute bacterial meningitis: diagnosed on detection of bacteria by CSF microscopy or culture, or acute clinical presentation with characteristic CSF findings (neutrophil predominance, raised protein, reduced glucose), and prompt response to antibacterial drugs [2, 3].

Patients were excluded if records of their diagnostic information were incomplete or unavailable. Recurrent episodes of meningitis that occurred in patients after discharge from hospital during the follow-up period were included.

This was a descriptive observational study using frequencies and percentages to summarize categorical data and means, medians, and ranges to summarize continuous data. The SAPiT trial was approved by the Biomedical Research Ethics Committee at the University of KwaZulu-Natal and the South African government's Medicines Control Council.

## 3. Results

From 642 TB-HIV coinfected patients enrolled, 20 (3.1%) patients presented with clinical features suggestive of meningitis, with 10 (50%) classified as not having meningitis (two cerebral tuberculomas, one cerebral toxoplasmosis, three with normal CSF, and four with insufficient data to support our case definitions). Fourteen episodes of meningitis that met our case definitions occurred in 10 patients, summarized in Table 1.

Among the patients analyzed, the mean (range) age was 34 (24–48) years and 4 patients were female. The mean (range) BMI was 22.13 (15.9–30.2) kg/m^2^ and median (range) baseline CD4+ cell count was 13 (2–298) cells/mm^3^. For 8 patients, the episode of meningitis under study was the first Acquired Immunodeficiency Syndrome (AIDS) illness experienced. Five patients reported a previous history of TB and 2 reported a previous history of meningitis, both receiving antifungal prophylaxis for cryptococcal meningitis after the first episode.

The most common combination of presenting clinical features was headache and neck stiffness, seen in 11 (78.6%) episodes and the triad of headache, vomiting, and neck stiffness observed in 5 (35.7%) episodes. Only 2 patients presented with fever.

There were nine (9/14) episodes of cryptococcal meningitis (one with disseminated cryptococcal disease, Patient 3), three (3/14) episodes of tuberculous meningitis (one concomitantly with suspected neurocysticercosis, patient 9), and two (2/14) cases of meningitis with mixed aetiology (Patient 3, acute bacterial and cryptococcal; Patient 7, cryptococcal and tuberculous).

TB sputum culture testing was done for 8/10 patients with meningitis: 4 of these sputum samples were taken within 6 weeks of the diagnosis of meningitis. All 4 sputum samples were culture positive for TB and occurred in patients with cryptococcal meningitis. There were no cases of drug-resistant tuberculosis.

The median number of days (range) to the episode of meningitis from antituberculous therapy initiation was 109 (32–486) and from ART initiation in the 7 patients receiving ART was 70 (7–214) days. All patients who were diagnosed with tuberculous meningitis were already on antituberculous therapy with 2 patients in the last month of the continuation phase. Patients 7 and 8 (Table 1) received steroids as part of tuberculous meningitis management. Four patients (Patients 1, 3, 7, 10) had suspected immune reconstitution inflammatory syndrome (IRIS) based on the temporal association of meningitis to ART initiation.

Of the 10 patients, 7 died, with 4 deaths directly due to meningitis: cryptococcal infection, 1, tuberculosis, 2, and 1 patient with mixed cryptococcal and tuberculous meningitis. From patients receiving ART, four (4/7) died. The case fatality rate for cryptococcal meningitis alone was 11.1% (1/9) and increased to 22.2% (2/9) when cryptococcal meningitis contributed to death as part of a mixed infection. Among the 7 patients with cryptococcal meningitis, 3 had relapsing episodes. The case fatality rate for tuberculous meningitis alone was 66.6% (2/3) and increased to 75% (3/4) when tuberculous meningitis contributed to death as part of a mixed infection.

The distribution of deaths among the SAPiT arms was as follows: 2 deaths in the early arm (1 from meningitis); 2 deaths in the postintensive arm (1 from meningitis); 3 deaths in the postcontinuation arm (2 from meningitis). Three patients died within 3.5 months after the episode of meningitis from other causes, attesting to the advanced immunocompromised state of these patients.

## 4. Discussion

In this first case series evaluating meningitis in confirmed HIV-TB coinfected patients, we demonstrate that the leading cause of meningitis is cryptococcus followed by tuberculosis, which is in keeping with population-based reports from sub-Saharan Africa investigating meningitis in patients with HIV alone [2]. In this sample of coinfected patients, development of meningitis was associated with poor outcomes including relapsing meningitis (30%) and high mortality (40%), even in patients already initiated on ART (57%). Patients in this study were severely immunocompromised with low CD4+ cell counts (median 13 cells/mm^3^), which could account for the reduced frequency of fever as a presenting complaint and the high mortality and relapse rates.

The burden of tuberculous meningitis has escalated as HIV disease progression increases extrapulmonary tuberculous risk. As established previously [2], the proportion of tuberculous meningitis is commonly underestimated due to its associated diagnostic challenges, specifically the inability to isolate mycobacteria in the CSF [2, 4, 8]. In this study, microbiologic diagnosis of tuberculous meningitis was further compromised as patients had already received several weeks to months of antituberculous therapy. Our study highlighted the important occurrence of tuberculous meningitis in patients already receiving antituberculous therapy which, to our knowledge, has only been investigated once previously, also in Southern Africa [5]. Antituberculous drug resistance and TB-associated IRIS should be principal considerations in such patients. Coinfected patients had poorer outcomes with case fatality rates of 66.7% compared to a case fatality rate of 30% from reports of tuberculous meningitis patients without coinfection [4]. This highlights the need to reevaluate management of tuberculous meningitis particularly in patients already exposed to antituberculous therapy. Evaluation of alternate drugs that optimally penetrate the blood brain barrier and the timing and overall benefit of steroids in HIV-associated tuberculous meningitis remains a challenge. Isoniazid and pyrazinamide are first-line antituberculous drugs with good CSF penetration. Patients who develop tuberculous meningitis while in the continuation phase of antituberculous therapy are managed with the retreatment regimen which includes streptomycin. However, injectable aminoglycosides have poor distribution in the CSF. The use of second-line drugs or alternative agents with good CSF penetration such as cycloserine and ethionamide rather than the conventional retreatment regimen needs further investigation [9, 10].

The estimated case fatality rate of cryptococcal meningitis in sub-Saharan Africa of 70% is considerably higher than in our study [11]. However, our sample is small resulting in an unstable estimate. Other contributing factors that could account for this differential mortality include availability of dual systemic antifungal agents, the urban location of our research facility with established systems for rapid triage into appropriate care and supporting laboratory infrastructure.

Three of seven (42.9%) patients experienced cryptococcal meningitis relapses, exceeding relapse rates reported in patients with HIV alone (23%). Contributing factors to the high relapse rate include inadequate clinical and serological screening for cryptococcal disease with use of appropriate prophylaxis, fluconazole resistance, and cryptococcal associated IRIS in patients on ART [12]. The underlying pathophysiologic mechanisms for the higher rates of relapse in coinfected patients needs further study.

We acknowledge several limitations in this study, particularly the small sample size. Patients were admitted to various facilities and were managed by health care workers with differing skills and access to laboratory and pharmacological resources. As this was a retrospective chart review, the quality and completeness of data obtained varied between patients.

Meningitis in HIV-infected individuals can be caused by various aetiological agents [2]. This study demonstrates cryptococcus as the leading cause of meningitis in HIV-TB coinfected patients, highlighting the occurrence of meningitis due to causative agents other than TB, in HIV-infected patients with sputum culture positive TB. However, clinicians should be wary that tuberculous meningitis can occur in patients already receiving antituberculous therapy. Coinfected patients who developed cryptococcal meningitis demonstrated a higher relapse rate. The mortality from meningitis in coinfected patients is high, particularly in those with tuberculous meningitis, and even in patients on antiretroviral therapy, emphasizing the necessity for timely diagnosis of HIV and meningitis and to establish optimum timing of ART in patients with central nervous system infections. Our data supports the need for further research into strategies for early detection of meningitis, improved diagnostic methods for tuberculous meningitis, and evaluation of more effective drug regimens to improve the prognosis of HIV associated meningitis.

## Figures and Tables

**Table 1:** Baseline characteristics and details of meningitis in 10 patients coinfected with HIV and TB.

Patient	Age, years	Gender	SAPiT arm	CD4+ cell count, cells/mm^3^	First AIDS defining condition	Previous TB	Previous meningitis	Aetiology of meningitis	Recurrent Meningitis^a^ (time from first episode, days)	ART	Presenting clinical features	Outcome
1	31	F	Early	65	Yes	Yes	No	Cryptococcal	No	Yes	Headache, neck stiffness, photophobia	Recovered

2	30	F	Postcontinuation	298	Yes	No	No	Cryptococcal	No	Yes	Headache, neck stiffness, vomiting, photophobia	Recovered

3	45	F	Early	2	Yes	Yes	No	Cryptococcal & acute bacterial	No	Yes	Headache, neck stiffness, photophobia, confusion	Recovered

								Cryptococcal	Yes— cryptococcal (39 days)	Yes	Cutaneous cryptococcal lesions	Recovered

4	48	M	Postcontinuation	54	Yes	No	No	Cryptococcal	No	No	Headache, fever	Recovered, death from cerebral glioma

5	33	M	Postintensive	12	Yes	No	No	Cryptococcal	No	No	Headache, neck stiffness, vomiting	Recovered

								Cryptococcal	Yes—cryptococcal (40 days)	No	Headache, neck stiffness vomiting	Recovered, death from lung abscess

6	33	M	Postcontinuation	2	Yes	Yes	No	Cryptococcal	No	Yes	Headache, neck stiffness, confusion	Death

7	24	M	Postintensive	18	Yes	No	No	Cryptococcal	No	No	Headache, neck stiffness, vomiting	Recovered
								Cryptococcal	Yes—cryptococcal (126 days)	Yes	Headache, neck stiffness, vomiting, fever	Recovered with neurological impairment
								Cryptococcal & Tuberculous	Yes—Tuberculous and cryptococcal (188 days)	Yes	Headache, neck stiffness, fever, focal signs	Death

8	34	M	Early	8	Yes	No	No	Tuberculous	No	No	Headache, neck stiffness, photophobia, loss of appetite	Recovered, death from pneumonia

9	33	M	Early	7	No	Yes	Yes	Tuberculous	No	Yes	Headache, neck stiffness, confusion, focal signs	Death

10	26	F	Postcontinuation	14	No	Yes	Yes	Tuberculous	No	Yes	Neck stiffness, confusion	Death

^a^Recurrent meningitis refers to cases of meningitis that occurred during the follow-up period.

*Note*. All cases of cryptococcal meningitis were confirmed by a positive India ink stain or positive cryptococcal antigen test on CSF. The diagnoses of tuberculous meningitis were based on clinical features and CSF abnormalities in keeping with our case definition. *Mycobacterium tuberculosis *was not isolated in the CSF in any of these cases.

## References

[1] Lawn SD, Churchyard G (2009). Epidemiology of HIV-associated tuberculosis. *Current Opinion in HIV and AIDS*.

[2] Jarvis JN, Meintjes G, Williams A, Brown Y, Crede T, Harrison TS (2010). Adult meningitis in a setting of high HIV and TB prevalence: Findings from 4961 suspected cases. *BMC Infectious Diseases*.

[3] Bergemann A, Karstaedt AS (1996). The spectrum of meningitis in a population with high prevalence of HIV disease. *Quarterly Journal of Medicine*.

[4] Thwaites GE, Bang ND, Dung NH (2005). The influence of HIV infection on clinical presentation, response to treatment and outcome in adults with tuberculous meningitis. *Journal of Infectious Diseases*.

[5] Silber E, Sonnenberg P, Ho KC (1999). Meningitis in a community with a high prevalence of tuberculosis and HIV infection. *Journal of the Neurological Sciences*.

[6] Katrak SM, Shembalkar PK, Bijwe SR, Bhandarkar LD (2000). The clinical, radiological and pathological profile of tuberculous meningitis in patients with and without human immunodeficiency virus infection. *Journal of the Neurological Sciences*.

[7] Abdool Karim SS, Naidoo K, Grobler A (2010). Timing of initiation of antiretroviral drugs during tuberculosis therapy. *New England Journal of Medicine*.

[8] Bhigjee AI, Padayachee R, Paruk H, Hallwirth-Pillay KD, Marais S, Connoly C (2007). Diagnosis of tuberculous meningitis: clinical and laboratory parameters. *International Journal of Infectious Diseases*.

[9] World Health Organization Treatment of tuberculosis: guidelines, 4th edition. http://www.who.int/tb/publications/cds_tb_2003_313/en/.

[10] Donald PR (2010). Cerebrospinal fluid concentrations of antituberculosis agents in adults and children. *Tuberculosis*.

[11] Park BJ, Wannemuehler KA, Marston BJ, Govender N, Pappas PG, Chiller TM (2009). Estimation of the current global burden of cryptococcal meningitis among persons living with HIV/AIDS. *AIDS*.

[12] Jarvis JN, Meintjes G, Williams Z, Rebe K, Harrison TS (2010). Symptomatic relapse of HIV-associated cryptococcal meningitis in South Africa: the role of inadequate secondary prophylaxis.. *South African Medical Journal*.

